# Aerobic fitness and fine motor skills are related to switching and updating in typically developing children

**DOI:** 10.1007/s00426-022-01749-w

**Published:** 2022-10-20

**Authors:** Stephanie Klupp, Alexander Grob, Wenke Möhring

**Affiliations:** 1 Department of Psychology, University of Basel, Missionsstrasse 62, 4055 Basel, Switzerland; 2 Department of Educational and Health Psychology, University of Education Schwäbisch Gmünd, Schwäbisch Gmünd, Germany

## Abstract

Movement is essential for everyday life and closely related to cognitive skills. The aim of the current research was to investigate whether different aspects of physical activity, i.e., aerobic fitness and motor skills, contribute above and beyond each other to the variance in children’s executive functioning. Children aged 8–13 years (*N* = 129, 58 females, *M*_age_ = 10.7 years, *SD*_age_ = 1.6 years) participated in the current cross-sectional study. Aerobic fitness was assessed by the Progressive Aerobic Cardiovascular Endurance Run (PACER). Motor skills were assessed using the standardized Movement Assessment Battery for Children 2nd edition (M-ABC-2), including fine motor skills, balance skills, and object control. Components of executive functions (inhibition, switching, updating) were assessed using the following tasks: an animal Stroop task, a local–global task, and a 2n-back task. Hierarchical regressions were conducted to analyze the relative importance of aerobic fitness and motor skills for children’s executive functions. Results indicated that aerobic fitness and fine motor skills were significantly related to switching and updating, whereas relations to inhibition were non-significant. Furthermore, it was found that fine motor skills explained additional variance above aerobic fitness in switching and updating whereas aerobic fitness did not add additional variance above fine motor skills in switching and updating. Balance and object control skills were not related to the three core executive functions. Results support the notion that aerobic fitness and fine motor skills are differently related to executive functions and highlight the importance of considering multiple components of constructs in future research.

## Introduction

Movement is an essential part of everyday life and is closely related to cognitive, social and emotional development (Libertus & Hauf, 2017; Mancini et al., 2016; Pesce et al., 2016). Besides several beneficial effects on brain functions, movement is also related to physical health (Hillman et al., 2008), as an active lifestyle is a protective factor against obesity, diabetes, and cardiovascular diseases and its importance increases across children’s development (Khan & Hillman, 2014). However, according to the World Health Organization (2020), 81% of adolescents do not meet the daily recommendations of at least 60 min moderate-to-vigorous-intensive physical activity across the week. This has also been shown in the SOPHYA study: 36% of Swiss children aged 6–16 years did not meet the recommended amount of daily movement (Bringolf-Isler et al., 2016). In addition, approximately 19% of children and adolescents in Switzerland are overweight (Bundesamt für Gesundheit, 2022). These prevalence rates underline the importance of understanding the effects of movement on children’s cognitive skills.

Previous research has demonstrated that physical activity is specifically linked to executive functions (Khan & Hillman, 2014). Executive functions are top–down processes of cognitive control which facilitate goal-directed behavior and are associated with other cognitive functions, social behavior and motor development (for a review, see Diamond & Ling, 2019). This construct consists of three related but separable core components: inhibitory control, cognitive flexibility and working memory updating (Diamond, 2013; Diamond & Ling, 2019; Miyake et al., 2000). First, inhibitory control, also called inhibition, includes aspects such as self-control and selective attention, and is the ability to suppress an automated, predominant response (Diamond, 2013; Miyake et al., 2000). Second, cognitive flexibility is also known as set shifting or task switching and is the ability to change perspectives or adjust to changing demands (Diamond & Ling, 2019) or switch between a set of rules based on a cue (Miyake et al., 2000). Third, working memory updating (short: updating) is the process which allows to manipulate information held within working memory by evaluating and replacing old, no longer relevant information with newer, more relevant information (Baddeley et al., 1997; Miyake et al., 2000). The present study aims to clarify the relative importance of different aspects of children’s physical activity (i.e., aerobic fitness, motor skills) on children’s executive functioning. Importantly, the current study will take a holistic approach and will include all components of this multifaceted construct of executive functions. Such an approach is crucial for creating informed interventions and will increase the understanding of how exactly motor and cognitive development are related.

The idea that motor and cognitive skills are closely related has a long history in psychological theories. For example, Piaget suggested that sensorimotor experiences in the first years of life, such as the integration of sensory inputs with motor actions, contribute to the understanding of the surrounding world (Piaget & Cook, 1952) which thereupon enhances cognitive development. The link between motor skills and children’s cognitive development can further be explained by the embodied cognition theory. According to this theory, motor skills facilitate the interactions with objects and other individuals which in turn promote cognitive development (Barsalou, 1999; Leonard, 2016; Loeffler et al., 2016; Pesce et al., 2016). The close link between motor and cognitive skills is also supported by similar developmental trajectories of motor skills and executive functions. Just like executive functions, selected motor skills such as fine motor skills appear to follow a lengthy developmental path and develop well into adolescence (Diamond, 2000). In addition, motor control and executive functions are accompanied by co-activation of certain brain regions, such as the prefrontal cortex, the cerebellum and the basal ganglia (Diamond, 2000).

Researchers have proposed several mechanisms underlying the relation between motor development and cognition. One set of mechanisms refers to long-term physiological changes in the brain after aerobic exercise. Intervention studies demonstrated beneficial effects on executive functions after chronic interventions (Liu et al., 2020; Verburgh et al., 2014). These chronic physiological changes refer to increased levels of brain-derived neurotrophic factors which facilitate synaptic plasticity. Such neurotrophic factors contribute to the growth of neurons and support learning and memory functions (Phillips, 2017). Further, chronic physical exercise influences the neuronal systems of attention, learning and memory as it increases neuroelectric activity, brain volume and blood flow, allowing for more efficient and flexible cognitive functioning (Ratey & Loehr, 2011). In addition, cognitive engagement during physical activity may depend on the complexity of the movement, with more complex motor tasks involving higher cognitive engagement which may have stronger effects on executive functions (Best, 2010; Ludyga et al., 2020; Pesce, 2012).

## Aerobic fitness and executive functions

It is important to define and consider the different aspects of human movement as this will help integrate previous research findings. Although physical activity and physical fitness are aspects of human movement and closely related to one other, they are conceptually distinct. While physical activity is a behavior that is defined as “any bodily movement produced by skeletal muscles that results in energy expenditure” (Caspersen et al., 1985, p. 126, p. 129); physical fitness is a physiological attribute that is defined as the ability to perform human movements such as physical activity (Caspersen et al., 1985; Pettee Gabriel et al., 2012). Physical fitness refers to an individual’s capacity of cardiovascular and respiratory systems to utilize oxygen, as well as the ability to carry out lengthy vigorous exercise (Esteban-Cornejo et al., 2019; Ortega et al., 2008), and thus creates a crucial interplay with physical activity. Further, physical fitness consists of various skill- and health-related components such as coordination, balance, muscular endurance and strength, flexibility as well as cardiorespiratory endurance which is often also called aerobic fitness (Caspersen et al., 1985; Pettee Gabriel et al., 2012).

Research investigating associations between children’s aerobic fitness and executive functioning yielded close associations. Studies have rarely assessed all three components of executive functions in a single study design (for an overview, see van Waelvelde et al., 2019), however, a recent review summarized several single studies and reported a positive, cross-sectional association, with greater aerobic fitness being related to children’s increased executive functioning (van Waelvelde et al., 2019). However, when focusing on particular relations between aerobic fitness and the three core executive functions, results are less consistent. For example, Nieto-Lopez et al. (2020) found significant relations between aerobic fitness and inhibition, but no significant relation to switching (updating was not investigated). Pindus et al. (2016) demonstrated neither significant associations between aerobic fitness and inhibition nor working memory (switching was not investigated). Possible explanations for the inconsistency may refer to using different measures and the assumed impurity of executive functions tasks. For example, Zhan et al. (2020) have found significant associations between aerobic fitness and response times for all three executive functions, however, when using accuracy as a dependent variable only the relation to updating was significant.

## Motor skills and executive functions

Another aspect of movement includes motor skills, which are defined as goal-directed movement patterns including running, throwing and writing (Burton & Rodgerson, 2001). Motor skills vary widely among individual children and will predict children’s physical activity. From a theoretical point of view, motor skills are a complex construct that can be separated into several subcomponents. Naturally, such a view influences standardized assessment. In several standardized assessments, general motor ability is separated into fine motor skills, balance skills, object control skills and locomotion (Gandotra et al., 2021; Henderson et al., 2007). Fine motor skills relate to the control of small muscle movements using hand–eye coordination (Clark & Whitall, 1989; Magill, 1996). Balance skills refer to the ability to maintain equilibrium while standing or moving (Caspersen et al., 1985). Object control skills involve the control of objects such as balls with either the hand or the foot, including for example catching, throwing or kicking (Clark & Whitall, 1989; van Capelle et al., 2017). Locomotion is defined as the movement of the body from one point to another by means of walking, running or jumping (Clark & Whitall, 1989; van Capelle et al., 2017).

The relation between motor skills and children’s executive functions was historically often examined in children with developmental coordination disorder or attention deficit hyperactivity disorder due to a high comorbidity between these pathologies. A first systematic review on the relation between motor and cognitive skills that focused on typically developing children was published by van der Fels et al. (2015). Regarding executive functions, the authors showed moderate-to-weak correlations with fine motor skills, moderate-to-no correlations with object control, and weak-to-no correlations with locomotion, while correlations with balance skills were not presented (van der Fels et al., 2015). The conclusions drawn from this review revealed an insufficient number of studies examining this relation between subcomponents of motor skills and children’s cognition but tentatively suggested that more complex motor skills seemed related to higher-order cognitive functions. Following this review, the number of publications increased demonstrating significant relations between executive functions with fine motor skills (e.g., Oberer et al., 2017; Stockel & Hughes, 2016), as well as gross motor skills (e.g., Stuhr et al., 2020; van der Fels et al., 2019). In addition, a recent meta-analysis examined the relation between motor skills and executive functions more closely. This meta-analysis by Gandotra et al. (2021) confirmed significant and robust effects between fine motor skills and all three executive function components. However, there was no significant relation between object control and executive functions, with low Rosenthal’s fail-safe-*N* values indicating no robust results. In addition, locomotion skills revealed a significant and robust effect with working memory, while inhibition and switching were not significantly related to locomotion and Rosenthal’s fail-safe-N revealed the findings must be interpreted with precaution. In addition to the review from van der Fels et al. (2015), significant associations between balance skills and all three executive functions were found, with only inhibition reaching a robust effect (according to Rosenthal’s fail-safe-*N*).

## The present study

Building on the previous studies above, the aim of the current research was to examine how different aspects of physical activity, namely aerobic fitness and motor skills, relate to all three core subcomponents of executive functions. In extension to previous studies, these associations were not only considered separately but also simultaneously. Thus, the present study investigated whether aerobic fitness and motor skills would contribute above and beyond each other to the variance of children’s executive functioning. This approach is innovative because it will increase the in-depth understanding of the specific relationships between the variables of interest. Previous literature is qualified by (a) investigating all three subcomponents of executive functions allowing a comprehensive overview; (b) exploring the relative importance of motor skills and aerobic fitness on children’s executive functions; and (c) controlling for important confounding variables.

With respect to the first point, published articles examining these relations have seldomly included all three core executive functions but rather focused on one or two. For example, 19 of the 26 studies in the review about physical fitness from van Waelvelde et al. (2019) examined one executive function, five studies examined two components and only two studies (Aadland et al., 2017; Schmidt et al., 2015) examined all three executive functions. Furthermore, it was reported that in particular switching was underrepresented and examined in only seven of the 26 studies. Furthermore, looking at executive functions individually is important for at least two reasons. On one hand, theoretical models advocate the separability of the components (Diamond, 2013) and findings may yield crucial information with respect to this point. On the other hand, studies show that the three components reveal differences in their developmental trajectories, such that inhibition for example seems to develop first out of the three (Huizinga et al., 2006). Given that 8- to 13-year-olds were included in the present study, findings may add to our understanding about these different developmental paths.

With respect to the second point, the literature highlights that aerobic fitness and motor skills are both related to executive functions; however, there is also evidence suggesting that they may be differently related. In the review from Haapala (2013), aerobic fitness seemed associated with tasks that require memory encoding, while motor skills seemed closely related to inhibitory control. In addition, many studies examining the relation between motor skills and executive functions did not control for aerobic fitness (e.g., Houwen et al., 2017) and vice versa (e.g., Scudder et al., 2014), although the relation between motor skills and aerobic fitness is well-known (e.g., Lubans et al., 2010).

Regarding the last point, many studies included in the meta-analysis by Gandotra et al. (2021) did not report information regarding participants’ socioeconomic status. Furthermore, other confounding variables such as sex, body mass index or intelligence were not consistently accounted for within the studies. Therefore, the current study examined relations among motor skills, aerobic fitness, and executive functions whilst accounting for age, sex, body mass index, intelligence and parental education (as an indicator for socioeconomic status). Building on the body of research outlined above, it is hypothesized that motor skills and aerobic fitness explain significant variance in children’s executive functioning (inhibition, switching, and updating). As previous research does not allow explicit expectations, it is further explored whether aerobic fitness and motor skills would contribute above and beyond each other to the variance of children’s executive functioning (in each separate component).

## Materials and methods

### Participants

One hundred and thirty-nine children aged 8–13 years participated in the current cross-sectional study examining motor and cognitive skills (for demographic details, see Table 1). Children were recruited from 57 local schools and fulfilled the inclusion criteria of no developmental or psychopathological diagnosis (e.g., attention deficit hyperactivity disorder) according to a parental questionnaire. The local ethics committee approved the present study. Prior to participation, parents signed a written informed consent, while children assented verbally.

**Table 1:** Descriptive statistics of key variables (*N* = 129)

Variable	*N* (%) / *M* (*SD*)	Range
Age (in years)	10.73 (1.60)	8–13
Sex		
Male	71 (55%)	
Female	58 (45%)	
Body mass index^a^	17.57 (2.34)	14–26
Intelligence^b^	114.26 (15.60)	72–156
Parental education	6.05 (1.34)	1–7
No school degree	2 (1.6%)	
Primary school	0 (0%)	
Mandatory school	1 (0.8%)	
Apprenticeship	22 (17.1%)	
High school	9 (7%)	
Higher education	22 (17.1%)	
University/College	73 (56.6%)	
Aerobic fitness (PACER)^c^	41.34 (16.32)	10–94
Fine motor skills (M-ABC-2)^d^	9.19 (2.32)	4–15
Balance skills (M-ABC-2)^d^	12.15 (2.41)	5–15
Object control skills (M-ABC-2)^d^	10.48 (2.57)	4–15
Inhibition errors (animal Stroop)	4.38 (3.45)	0–15
Switching errors (local–global)	10.15 (6.90)	0–32
Updating errors (2n-back)	10.25 (9.76)	0–42

Data are presented as absolute (and relative) frequencies or means (*SD*)

^a^Body mass index is calculated by the formula of kilograms x height in meters squared

^b^Intelligence was computed in accordance with recommendations from Waldmann (2008) using the four subtests vocabulary, matrix reasoning, letter-number sequencing, and coding of the Wechsler Intelligence Scale for Children 4th edition (WISC-IV; Petermann & Petermann, 2011)

^c^Aerobic fitness is determined by the number of laps in the Progressive Aerobic Cardiovascular Endurance Run (PACER; Meredith & Welk, 2010)

^d^Motor skills are measured with the standardized Movement Assessment Battery for Children, 2nd edition (M-ABC-2; *M* = 10, *SD* = 3, range 1–19; Henderson et al., 2007)

### Measures and procedure

To examine the relation between different aspects of physical activity (motor skills, aerobic fitness) and executive functions, children were individually assessed in two two-hour sessions in two consecutive weeks at the laboratory of the University of Basel. In the first session, participants’ anthropometric data (e.g., height, weight) was measured and they solved a series of executive function tasks. At the end of this session, children performed a Progressive Aerobic Cardiovascular Endurance Run (PACER; Léger et al., 1988). Within this first session, participants also performed several dual tasks that are described elsewhere and are beyond the scope of the present study (Möhring et al., 2020). The second assessment session included standardized batteries such as the Movement Assessment Battery for Children 2nd edition (M-ABC-2; Henderson et al., 2007). To account for children’s intellectual functioning, they also solved four subtests (vocabulary, matrix reasoning, letter-number sequencing, and coding) of the German version of the Wechsler Intelligence Scale for Children 4th edition (WISC-IV; Petermann & Petermann, 2011). Performance on these subtests was computed to an intelligence score in accordance with recommendations from Waldmann (2008). In addition, parents filled in a questionnaire including demographic and general information about their child and their education, which were included as control variables in the statistical analysis.

### Aerobic fitness assessment

The PACER measures physical fitness and more specifically aerobic capacity (for a review, see van Waelvelde et al., 2019). It is an established reliable and valid measure in children and adolescents (Carrel et al., 2012; Olds et al., 2006). Due to limited room length, the shortened version was used, consisting of two borders 15 m apart (Meredith & Welk, 2010). Participants were asked to run back and forth between these borders as long as possible. An audio recording with sound signals indicated when participants should have reached the opposite border. These signals set the pace by starting slowly and increasing progressively. The shuttle run was finished when the participant failed to reach a border before the signal for a second time. The score composed of the number of completed runs, with a greater score indicating a higher level of aerobic capacity.

### Motor skill assessment

Motor skills in children aged 3.0–16.11 years can be measured with the M-ABC-2 on the three motor subcomponents (a) manual dexterity indicating fine motor skills; (b) balance skills and (c) aiming and catching also labeled as object control skills. This standardized motor assessment reports high retest-reliability coefficients between 0.73 and 0.84 and high inter-rater reliability coefficients between 0.92 and 1.00 (cf. the M-ABC-2 manual; Henderson et al., 2007, p. 139). The tasks differ slightly for the two age bands of 7–10 years and 11–16 years. Most tasks included practice attempts before testing. Furthermore, most tasks included two attempts for each task of which the better score was taken into account.

The subcomponent fine motor skills consists of three subtests: a one-handed posting task, a bimanual assembly task, and a trail-drawing task. The younger age group placed pegs into a board, threaded a lace, and drew a line through a trail. The older age group turned pegs on a board, constructed a triangle with nuts and bolts, and drew a line through a narrower trail. While time of completion was assessed in the first two subtests, the third subtest measured the number of errors.

The subcomponent balance consists of three subtests: a board balance task, a walking balance task and a jumping task. The younger age group completed a one-legged stance on a balance board, a heel-to-toe forwards walk, and a one-legged hopping task. The older age group completed a heel-to-toe stance on a balance board, a toe-to-heel-backwards walk and one-legged zigzag hopping task. The first task was measured in seconds; the second task was measured by the number of successful steps; the third task by the number of successful jumps.

The subcomponent object control consists of two subtests: catching a ball and aiming at a target. The younger age group was asked to throw a tennis ball against a wall and catch it with both hands as well as to throw a bean bag onto a target mat on the floor. The older age group was asked to catch the ball with one hand only and throw the ball one-handed at a target on the wall. Both tasks were completed once with the left and once with the right hand and the number of successful completions were measured.

Raw scores of the subtests were converted into age-standardized scores, resulting in scores for the three subcomponents (fine motor skills, balance, object control) as well as the total motor score (*M* = 10, *SD* = 3, range 1–19). The total motor score is often used in clinical samples but also in typically developing samples to indicate motor problems such as developmental coordination disorder (Houwen et al., 2017; Kaiser et al., 2015). The equivalent percentile of the total motor score suggests a significant motor difficulty below the 5^th^ percentile and a risk of motor difficulty below the 16th percentile (Sartori, 2019).

### Executive function assessment

Participants completed three tasks each tapping one of the executive functions (inhibition, switching, and working memory updating). The order of the executive function tasks was counterbalanced across participants. Children were sitting on a chair with the stimuli being projected on a wall in front of them. The test phase for each executive function task included eight pre-randomized trials with a total of 96 items. Children were asked to say their answers out loud. The number of errors out of the maximum of 96 possible correct answers served as a dependent variable.

#### Adjustment

Generally, tasks measuring executive function components differ widely with respect to the presented material (e.g., visual, auditory stimuli), the required response (e.g., verbal, motor response), and their difficulty. In the current study, comparability across the executive function tasks was increased using visual material only, recording verbal responses, and equating difficulty across participants and executive function tasks. Such an adjustment controls for differences among individual children’s baseline performance (Saxena et al., 2017) and ensures that effects are not found because of inherent differences in difficulty among the executive function tasks. First, children were familiarized with each executive function task through practice trials. Then, participants completed a set of easy items (level 1) with long presentation times and long interstimuli-intervals, to identify their baseline performance level. In a subsequent set of level-2 items, presentation times and interstimuli-intervals were shortened to decrease participants’ performance level to approximately 90% as compared to their baseline level-1. If children did not meet this criterion, participants completed another set of level-3 items with even shorter presentation times and inter-stimuli-intervals. Therefore, the dependent variable of the current study relates to the performance level, at which the participants met the criterion of approximately 90% performance. For example, if a participant produced five errors at baseline level-1, the participant would be expected to produce approximately ten errors in the more difficult level-2 and if not achieved, level-3 would be applied (for a more detailed description of the procedure, stimuli sizes and presentation times, see Möhring et al., 2020).

#### Inhibition

Participants completed a classic Stroop task as a measure of their ability to inhibit a predominant response (Stroop, 1935). An animal Stroop task was used in which the stimuli comprised of a picture of one of four animals used from the Intelligence and Development Scales-2 (Grob & Hagmann-von Arx, 2018). Children were asked to name the correct color of this animal (blue—dolphin, yellow—chick, green—frog, red—ladybug) as quickly and accurately as possible. In accordance to Miyake et al. (2000), the total of 96 items contained 48 neutral items with animals printed in black–white, 12 congruent items displaying animals in the correct color (e.g., a green frog), and 36 incongruent items displaying animals in the incorrect color (e.g., a blue frog).

#### Switching

Participants completed a local–global task as a measure of their ability to switch between a set of rules (Navon, 1977). Children were presented with a large, global figure that was composed of many smaller, local figures (e.g., a triangle made of many smaller circles). Stimuli consisted of four geometric shapes (circle, cross, triangle, square) which were systematically combined with the exception that the global figure could not be identical to the local figures. This resulted in 12 possible combinations. The cue indicating which figure to name was determined by the color it was presented in. When the figure was presented in blue color, children were asked to say the name of the global figure; when the figure was presented in black, they were asked to say the name of the local figure. While Miyake et al. (2000) instructed adults to name the number of lines of the target figure, the current task was adapted for children by asking them to name the geometric figure instead. Out of the 96 items total, 48 were presented in blue and black color, respectively. Furthermore, half of the trials involved no switch (no change in color), while the other half involved a switch (either from local to global or from global to local).

#### Updating

Participants completed a 2n-back task as a measure of their ability to update old, no longer relevant information with newer, more relevant information (Dobbs & Rule, 1989). Stimuli consisted of single digits from 1 to 9. Digits were presented in pre-randomized trials with two constraints. First, consecutive numbers did not occur and secondly, the identical digit did not repeat within a proximity of three positions. Children were asked to postpone the naming of each digit by two positions (2n-back; cf. Schaefer et al., 2008).

### Statistical analysis

One participant was excluded due to a diagnosis with attention deficit hyperactivity disorder as reported within the parental questionnaire and, therefore, not fulfilling an inclusion criterion. Another participant was excluded due to potentially being at risk for a Developmental Coordination Disorder (M-ABC-2 < 16th percentile; Henderson et al., 2007), while no child had to be excluded due to a potential intellectual impairment (WISC-4 IQ < 70; Petermann & Petermann, 2011). Furthermore, eight participants had to be excluded due to missing data in the key variables (M-ABC-2: *n* = 3; PACER: *n* = 1; WISC-4: *n* = 1; parental education: *n* = 3). Therefore, the final sample consisted of 129 typically developing children (58 females, *M*_age_ = 10.7 years, *SD*_age_ = 1.6 years, see Table 1 for demographic information).^1^ Analyses were performed using IBM SPSS 26.

To analyze the relative importance of motor skills and aerobic fitness for children’s executive functions, a series of hierarchical regressions was conducted. Regressions were computed separately for each of the three dependent variables of executive functions (inhibition, switching, updating). In the first step, the control variables age, sex, body mass index, intelligence and parental education were entered. In the second step, aerobic fitness was entered, followed by fine motor skills, balance skills and object control skills in the steps three to five respectively. The order of these motor skills was determined by the expected associations according to conclusions from a recent meta-analysis (Gandotra et al., 2021).^2^ Furthermore, variables were entered in separate steps to identify the amount of explained variance for each skill. Vice versa, this analysis was repeated in reversed order to also determine the explained variance of aerobic fitness above and beyond the motor skills. In this regression analysis, control variables were again entered in the first step, fine motor skills were entered in the second step, balance skills and object control skills in steps three and four, and last, aerobic fitness in the fifth step.

## Results

Descriptive statistics of demographic variables, aerobic fitness, motor skills and executive functions of children are provided in Table 1. Hierarchical regression analyses are presented in Tables 2 and 3. Results are accounted for effects of age, sex, body mass index, intelligence and parental education in the first step of the hierarchical regression. Effects of age were significant across all three executive functions (all *p*s < 0.001; all *ß*s > − 0.423), revealing that older children showed fewer errors than younger children in inhibition, switching, and working memory updating. Further, a significant sex effect was found for the inhibition task (*p* = 0.002; *ß* = 0.264), due to males making more errors; however, no significant sex effects were found in the switching or updating tasks (both *p*s > 0.125; both *ß*s < 0.114). In addition, the body mass index revealed a tendency in inhibition and switching (both *p*s < 0.074; both *ß*s > 0.154), but no significance in updating (*p* = 0.380; *ß* = 0.069), because children with a higher body mass index showed more errors in inhibition and switching. Additionally, intelligence was significantly related to switching and updating (both *p*s < 0.030; both *ß*s > − 0.177), but not to inhibition (*p* = 0.784; *ß* = − 0.023), indicating that children with higher intelligence showed fewer errors in the switching and updating task. Parental education was not significantly related to errors in executive functions (all *p*s > 0.154; all *ß*s < 0.115). Overall, the control variables explained a significant portion of the variance for inhibition (21.1%), switching (25.6%), and updating (35.8%; see Step 1 in Tables 2 and 3).

**Table 2:** Hierarchical regression analyses examining relations between aerobic fitness, motor skills, and executive functions (*N *= 129)

	Inhibition		Switching		Updating	
Model and variable	∆*R*^2^	*β*	∆*R*^2^	*β*	∆*R*^2^	*β*
Step 1	0.211***		0.256***		0.358***	
Age		– 0.423***		– 0.519***		– 0.554***
Sex		0.264**		0.041		0.114
Body mass index		**0.156**^**+**^		**0.154**^**+**^		0.069
Intelligence		– 0.023		– 0.177**		– 0.283**
Parental education		0.053		0.115		0.038
Step 2	0.007		0.040*		0.026*	
Age		– 0.365**		– 0.380***		– 0.442***
Sex		0.273**		0.064		0.132^**+**^
Body mass index		0.121		0.07		0.001
Intelligence		– 0.024		– 0.181*		– 0.285***
Parental education		0.06		**0.132**^**+**^		0.051
Aerobic fitness		– 0.101		– 0.244*		– 0.196*
Step 3	0.001		0.032*		0.026*	
Age		– 0.370**		– 0.416***		– 0.475***
Sex		0.265**		0.000		0.075
Body mass index		0.125		0.099		0.027
Intelligence		– 0.018		**– 0.137**^**+**^		– 0.246**
Parental education		0.058		0.119		0.040
Aerobic fitness		– 0.091		**– 0.168 + **		– 0.128
Fine motor skills		– 0.027		– 0.203*		– 0.183*
Step 4	0.001		0.004		0.003	
Age		– 0.370**		– 0.417***		– 0.474***
Sex		0.260**		0.013		0.064
Body mass index		0.119		0.114		0.014
Intelligence		– 0.018		**– 0.138**^**+**^		– 0.244**
Parental education		0.061		0.111		0.047
Aerobic fitness		– 0.087		**– 0.181**^**+**^		– 0.117
Fine motor skills		– 0.023		– 0.217*		– 0.171*
Balance skills		– 0.025		0.072		– 0.061
Step 5	0.000		0.003		0.000	
Age		– 0.366**		– 0.405***		– 0.472***
Sex		0.267**		0.034		0.067
Body mass index		0.117		0.107		0.013
Intelligence		– 0.017		**– 0.136**^**+**^		– 0.244**
Parental education		0.059		0.104		0.046
Aerobic fitness		– 0.084		**– 0.174**^**+**^		– 0.115
Fine motor skills		– 0.018		– 0.202*		– 0.169*
Balance skills		– 0.022		0.082		– 0.060
Object control skills		– 0.021		– 0.065		– 0.010

Variables are entered in a way answering the question whether motor skills explain additional variance above aerobic fitness on children’s executive functions

Step 1: model with control variables explaining performance in executive function. Step 2: Aerobic fitness was added. Step 3: fine motor skills were added. Step 4: Balance skills were added. Step 5: Object control skills were added. Sex: − 1 = female; + 1 = male

Significant results ^+^*p* < 0.1, *p < 0.05, **p < 0.01, ***p < 0.001 are presented in bold

**Table 3:** Hierarchical regression analyses examining relations between motor skills, aerobic fitness, and executive functions (*N* = 129)

	Inhibition		Switching		Updating	
Model and variable	∆*R*^2^	*β*	∆*R*^2^	*β*	∆*R*^2^	*β*
Step 1	0.211***		0.256***		0.358***	
Age		– 0.423***		– 0.519***		– 0.554***
Sex		0.264**		0.041		0.114
Body mass index		**0.156**^**+**^		**0.154**^**+**^		0.069
Intelligence		– 0.023		– 0.177**		– 0.283**
Parental education		0.053		0.115		0.038
Step 2	0.003		0.055**		0.042**	
Age		– 0.421***		– 0.510***		– 0.546***
Sex		0.249**		– 0.029		0.053
Body mass index		**0.156**^**+**^		0.157^+^		0.071
Intelligence		– 0.012		– 0.124		– 0.236**
Parental education		0.051		0.106		0.030
Fine motor skills		– 0.054		– 0.252**		– 0.220**
Step 3	0.001		0.002		0.005	
Age		– 0.417***		– 0.516***		– 0.537***
Sex		0.244**		– 0.022		0.042
Body mass index		0.146		0.171*		0.05
Intelligence		– 0.011		– 0.124		– 0.235**
Parental education		0.056		0.099		0.040
Fine motor skills		– 0.045		– 0.264***		– 0.201*
Balance skills		– 0.037		0.048		– 0.077
Step 4	0.001		0.005		0.000	
Age		– 0.410***		– 0.496***		– 0.532***
Sex		0.253**		0.006		0.049
Body mass index		0.142		0.159^+^		0.047
Intelligence		– 0.011		– 0.123		– 0.235**
Parental education		0.053		0.091		0.038
Fine motor skills		– 0.038		– 0.243***		– 0.196*
Balance skills		– 0.032		0.062		– 0.073
Object control skills		– 0.028		– 0.080		– 0.020
Step 5	0.004		**0.018**^**+**^		0.008	
Age		– 0.366***		– 0.405***		– 0.472***
Sex		0.267**		0.034		0.067
Body mass index		0.117		0.107		0.013
Intelligence		– 0.017		– 0.136^+^		– 0.244**
Parental education		0.059		0.104		0.046
Fine motor skills		– 0.018		– 0.202*		– 0.169*
Balance skills		– 0.022		0.082		– 0.060
Object control skills		– 0.021		– 0.065		– 0.010
Aerobic fitness		– 0.084		**– 0.174**^**+**^		– 0.115

Variables are entered in a way answering the question whether aerobic fitness explains additional variance above motor skills on children’s executive functions

Step 1: model with control variables explaining performance in executive function. Step 2: fine motor skills were added. Step 3: Balance skills were added. Step 4: Object control skills were added. Step 5: Aerobic fitness was added. Sex: − 1 = female; + 1 = male

Significant results ^+^*p* < 0.1, *p < 0.05, **p < 0.01, ***p < 0.001 are presented in bold

**Fig. 1:**
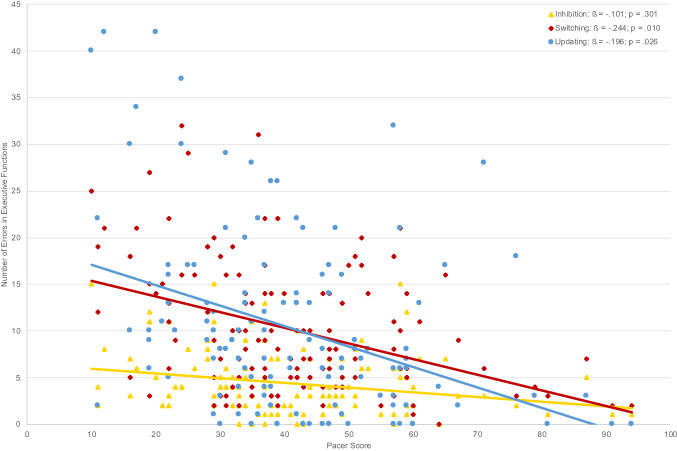
Relations between executive function with aerobic fitness (*N* = 129)

In the first series of hierarchical regressions (Table 2), aerobic fitness was added in the second step and explained a significant part of the variance in the switching task (4.0%, see Fig. 1). When entering fine motor skills in the third step of the same regression, another significant part of the variance in switching (3.2%) was explained. Similar results are found for the updating task: Entering aerobic fitness in step 2 explained a significant part of the variance in the updating task (2.6%; see Fig. 1), and another significant part of the variance in updating (2.6%) was explained through fine motor skills*.* Balance and object control skills did not explain any additional variance for switching and updating. The regression analysis with inhibition as a dependent variable revealed no significant relations. Therefore, it seems that fine motor skills explain variance in children’s switching and updating skills beyond aerobic fitness.

**Fig. 2:**
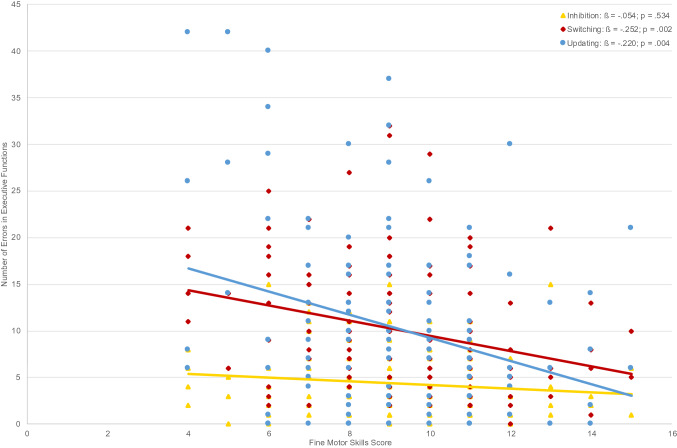
Relations between executive function with fine motor skills (*N* = 129)

In the second series of hierarchical regressions (Table 3), in which control variables were entered in the first step, followed by fine motor skills, balance, and object control in steps two to four, and aerobic fitness in the last step of the model, results showed an even clearer picture of the relative contributions. Adding fine motor skills in the second step explained a significant part of the variance in the switching task (5.5%, see Fig. 2). Entering aerobic fitness in the last step yielded a non-significant explained variance of 1.8% (*p* = 0.079) in switching. Similar results are found for the updating task: Adding fine motor skills in the second step explained a significant part of the variance in updating (4.2%; see Fig. 2), while adding aerobic fitness in the last step was not found to add any explained variance in updating (0.8%). Again, balance and object control skills did not add any explained variance in switching and updating. Moreover, no significant relations were found between motor variables and inhibition. Results indicate that aerobic fitness does not seem to explain any significant variance beyond fine motor skills.

## Discussion

The current study investigated the relations between children’s aerobic fitness, motor skills, and executive functions, whilst considering relevant confounding variables such as age, sex, body mass index, intelligence and parental education (e.g., Gandotra et al., 2021). In the hierarchical regression analyses, it was found that aerobic fitness and fine motor skills were significantly related to switching and updating, however, relations to inhibition were non-significant. More concrete, it was found that fine motor skills explained additional variance above aerobic fitness in switching and updating. In contrast, aerobic fitness did not significantly explain any additional variance in children’s switching and updating performance when simultaneously accounting for fine motor skills. Interestingly, balance and object control skills were not related to any of the three core executive functions.

Most of the current results are in line with previous research. The significant results between aerobic fitness as well as fine motor skills with switching and updating strengthen the findings of recent reviews and meta-analyses (Gandotra et al., 2021; van der Fels et al., 2015; van Waelvelde et al., 2019). In line with Cabral et al. (2021), a significant association between aerobic fitness and switching as well as a non-significant relation to inhibition was demonstrated. Furthermore, taking a closer look at the definition of motor skills above (Clark & Whitall, 1989), balance skills within the M-ABC-2 assessment do not only include components of balance but also locomotion such as jumping and walking forwards or backwards (referring to *dynamic* balance). The non-significant associations between balance skills and executive functions found in the current study are thus in accordance with conclusion from van der Fels et al. (2015), while Gandotra et al. (2021) found significant small effects to all three executive functions. However, given that Gandotra and colleagues suggested that the effects of switching and updating were not robust, the current results reinforce this interpretation of caution. Further, Gandotra et al. (2021) also found no significant effects between locomotion and inhibition and switching as well as between object control and all three executive functions which is in line with the present results.

Furthermore, previous research highlighted the importance of including control variables (Gandotra et al., 2021). The present analyses found females to produce fewer errors within the inhibition task compared to males which is supported by previous studies (Mileva-Seitz et al., 2015; Singh et al., 2022). However, a recent meta-analysis summarized findings from 22 studies and indicated no significant sex difference in inhibition, even though four studies showed differences in favor of females and two studies in favor of males (Gaillard et al., 2021). Similarly, the present findings regarding children with higher BMI producing more errors in inhibition and switching is in line with previous research (Lavagnino et al., 2016). The authors of this respective study explained the association as a lack of inhibitory control which may cause impulsive eating leading to obesity.

The main contradictory finding of the current study compared to the majority of previous studies are the non-significant relations to inhibition (for comparable results, see Aadland et al., 2017). From a descriptive perspective, this relation was similarly positive as for the switching and updating results, however, it was not significant. One possible explanation could be the examined age range of children. Studies that did find significant relations (e.g., Stockel & Hughes, 2016) have predominantly assessed children in kindergarten age (≤ 6 years of age; for an overview see, Gandotra et al., 2021). Building on research indicating that inhibition seems to develop at an earlier age as compared to switching and updating, it seems that relationships between motor skills and components of executive functions may change across childhood and adolescence (Best & Miller, 2010; Huizinga et al., 2006; Mohring et al., 2021). Another possible explanation could be the variety of tasks used in previous research. In the majority of studies, children performed a flanker task (e.g., Hillman et al., 2009; Roebers & Kauer, 2009; Scudder et al., 2014) or a Stroop task with numbers or fruits (e.g., Buck et al., 2008; Roebers et al., 2014; Van Der Veer et al., 2020). Other studies included tasks such as the Go/no-go task, Simon task, Cambridge neuropsychological test automated battery, neuropsychological assessment 2nd edition or parental questionnaires (for an overview see, Gandotra et al., 2021). However, there are studies that also used a similar animal Stroop task as in the current study (Wright et al., 2003), and found that children with poor fine motor skills exhibited lower inhibition skills (Stockel & Hughes, 2016). Last, another possible explanation could be the perceived difficulty of the present inhibition task. Even though there was considerable variation among children’s inhibition performance, children did show fewer errors in inhibition as compared to the other executive function tasks.

Regarding the findings showing that fine motor skills explain variance of children’s executive functions above aerobic fitness but not vice versa, there are different possible explanations. Previous research has suggested that aerobic fitness and fine motor skills are correlated (Haapala, 2013),^3^ and therefore, they reduce a part of the explained variance from each other. Interestingly, the present results suggest that this reduction is not the same strength in both directions. Tasks measuring fine motor skills often engage cognitive processes, such as for example decision making and sustained attention (Geertsen et al., 2016), whereas the aerobic fitness task is assumed to require less cognitive engagement (De Bruijn et al., 2020). These differences of cognitive demands in different types of movement are for example also found in current research of open vs. closed-skill sports, specifically in relation to executive functions (Formenti et al., 2021; Ludyga et al., 2022). Therefore, the differences in cognitive engagement inherent in each motor task could be one possible explanation for the different explained variance. Furthermore, a recent study examined the impact of task novelty on the relation between motor tasks and executive functions, indicating that newer tasks were more strongly related to executive functions than repeated known tasks (Maurer & Roebers, 2020). Given that the task measuring fine motor skills included three different measures as opposed to running back and forth between two borders in the aerobic fitness task, this aspect of novelty may have factored into the analyses. In addition, when looking at the repetitions within the two motor tasks, the majority of the tasks measuring fine motor skill were practiced twice before conducting two test trials. In contrast, the aerobic fitness task revealed a range of 10–94 runs, thus supporting the argument of higher task novelty within fine motor skills.

Moreover, the current study highlights that typically developing children with poor motor skills showed more errors in switching and updating tasks than children with higher motor skills. This result is particularly important for future interventions. These children with poor motor skills often do not meet the clinical cut-off values of motor difficulties as determined by criteria for a developmental coordination disorder according to the 5th edition of the Diagnostic and Statistical Manual (DSM-V). Other criteria such as from the Developmental Coordination Disorder Questionnaire (DCD-Q) indicate motor problems for scores below the 10th percentile and suspected motor problems between the 10th and 25th percentile, while the M-ABC-2 suggests a significant motor difficulty below the 5th percentile and a risk of motor difficulty below the 16th percentile (Sartori, 2019). However, the present results using a dimensional perspective indicate that even when children do not meet these criteria, they show significantly lower cognitive performance. This may hold not only for typically developing children but also for example for children with attention deficit hyperactivity disorder in which motor problems occur in 30–50% and are usually neither included in the assessment nor the intervention (Fliers et al., 2010; Klupp et al., 2021). Argumentatively, a more dimensional view onto children’s difficulties can be helpful in the future to accommodate and support also typically developing children with motor skills below average.

### Strengths, limitations and future research

The current study examined the relation between motor skills and executive functions in a large sample of school-aged children, and thus focused on an age range that has been insufficiently studied until now. The main strength of the current study is the integrated and differentiated approach by including several components of motor skills as well as children’s executive functions. The majority of previous studies has focused on only one or two executive functions, and in particular the switching component has often been neglected up to now (van Waelvelde et al., 2019). Therefore, findings of the current study could be strengthened in future research by taking care to cover several facets of executive functions and motor skills. Furthermore, it can be considered as a strength that aerobic fitness was assessed at the end of the test session and therefore after the executive function tasks. This order of presented tasks makes sure that there were no acute effects of physical fitness on performance in the executive function tasks. Regarding the assessment of motor skills, standardized and established measurements such as the M-ABC-2 and the PACER were conducted. In addition, common measures of executive functions were used such as the Stroop task, the local–global task and the 2n-back task.

There are limitations that warrant mention. Firstly, the animal Stroop task showed less errors as compared to the other executive function tasks which may indicate that the task might have not been challenging enough for the present sample. However, as can be seen in Table 1, there is still considerable variance within the task. Another possibility why inhibition did not reach significance as expected, might emerge from research showing that inhibition develops first out of the three executive functions (Huizinga et al., 2006). This may explain why studies with younger samples found significant relations (Stockel & Hughes, 2016) and why variances in the current study are greater in the other two executive function tasks. Future studies may examine other tasks tapping inhibition in samples of school-aged children, and preferably model each component of the executive functions (i.e., inhibition) as latent variables. Second, although the PACER is a standardized and established measure, fitness can also be measured using other assessments such as the maximal oxygen consumption (van Waelvelde et al., 2019). Ideally, future studies should include multiple assessments to form a latent construct. Third, the power analysis revealed that the present study is sufficiently powered. However, as other previous studies (for overviews, see Gandotra et al., 2021; van Waelvelde et al., 2019) have also found lower effect sizes, future studies should examine these associations with larger samples. In addition, the distribution of parental education within the present sample was slightly skewed towards higher-educated parents. Future studies should replicate the present findings using more representative samples. Lastly, the current study used cross-sectional data. This design precludes examining the direction of the effects as well as children’s intra-individual development. Thus, longitudinal research is needed and strongly recommended for future investigations.

## Conclusion

The present study revealed that fine motor skills explained additional variance above aerobic fitness in children’s switching and updating skills which could not be confirmed vice versa. This supports the notion that aerobic fitness and fine motor skills are differently related to executive functions (Haapala, 2013). Importantly, relations were only revealed for switching and updating, whereas inhibition showed no significant associations with motor skills nor aerobic fitness. In summary, the current findings emphasize the importance of considering multiple components of constructs such as aerobic fitness, motor skills and executive functions to ensure an overarching, comprehensive insight.

## Footnotes

1 Previous research has found small-to-moderate effect sizes in analyses showing that children’s fitness was associated with executive functions (van Waelvelde et al., 2019). A power analysis with G-Power 3.1, based on an effect size of *f*^2^ = 0.137 (e.g., from Buck et al., 2008), assuming a power of 0.80, and a significance level of *p* = .0.05, revealed a minimum sample size of 124 participants. Thus, it can be concluded that the present study should be sufficiently powered, using a final sample of 129 participants.

2 In additional analyses, the order of the three motor skills (fine motor skills, balance skills, and object control skills) in steps 3–5 were changed and no significant difference to the current results was found.

3 In the current study, children’s fine-motor skills and aerobic fitness were significantly correlated, *r* = 0.217, *p* < 0.05. Collinearity statistics revealed a diagnostic value of the variance inflation factor (VIF) of 1.12 which indicates no to low collinearity issues.
